# Factors Associated with the Quality of Life and Needs of Hemodialysis Patients in Saudi Arabia: A Basis for Improved Care

**DOI:** 10.3390/medicina61020180

**Published:** 2025-01-22

**Authors:** Talal Ali Hussein Alqalah, Gamil Ghaleb Alrubaiee, Sameer Abdulmalik Alkubati

**Affiliations:** 1 Department of Medical-Surgical Nursing, College of Nursing, University of Ha’il, Ha’il City 55476, Saudi Arabia; s.alkubati@uoh.edu.sa; 2 Department of Community Health Nursing, College of Nursing, University of Ha’il, Ha’il City 55476, Saudi Arabia; g.nasr@uoh.edu.sa; 3 Department of Community Health and Nutrition, Al-Razi University, Sana’a 12544, Yemen

**Keywords:** hemodialysis patient, quality of life, needs, predictors

## Abstract

*Background and Objectives*: End-stage renal disease threatens individuals’ quality of life (QoL) and has a substantial influence on their daily lives. While several studies have explored the QoL of hemodialysis patients, none have comprehensively examined the relationship between patient QoL and their specific needs as well as the factors that predict these outcomes. This study aimed to investigate the intricate relationship and predictive factors between QoL and the needs of hemodialysis patients, serving as a foundational step toward enhancing their care. *Materials and Methods*: This cross-sectional study included 222 patients undergoing hemodialysis for ESRD between January and March 2023. Data were gathered through structured interviews utilizing the World Health Organization Quality of Life-BREF, and questionnaires were needed. To assess intergroup differences, *t*-tests and ANOVA were employed, while multiple linear regression and correlation coefficients were used to unveil predictive associations. *Results*: Our findings revealed that the majority of patients interviewed expressed satisfaction with their health and reported high QoL. Predictors of higher QoL included being under 60 years of age, married, strong familial support, and a body mass index < 30 kg/m^2^. Conversely, living alone has emerged as a predictor of diminished QoL. Moreover, middle-aged patients who were married and had a normal BMI were identified as having lower needs. *Conclusions*: A discernible association was observed between higher QoL and reduced need among hemodialysis patients. This study offers valuable insights into the multifaceted factors influencing the QoL and needs of these patients, offering guidance for enhancing patient care and ultimately improving their overall QoL.

## 1. Introduction

End-stage renal disease (ESRD) is a formidable global health challenge characterized by irreversible degradation of kidney function [1,2], necessitating either dialysis or kidney transplantation for survival. Over the past three decades, the prevalence of ESRD has exhibited a consistent upward trajectory in the Arab world, including the Kingdom of Saudi Arabia, underscoring the importance of focused attention and targeted interventions. Currently, Saudi Arabia shoulders the care of over 20,000 dialysis patients, with 9810 receiving post-transplantation care [3]. Notably, the Saudi Center for Organ Transplantation reported an annual increase of 6% in the number of patients undergoing hemodialysis [4].

ESRD has a significant impact on patients, manifesting in profound physical, mental, and emotional repercussions that disrupt their daily lives [5]. Moreover, it exerts a substantial financial burden on patients, their families, and healthcare systems [6] as patients depend on renal replacement therapy for survival. Unfortunately, this treatment modality cannot fully compensate for the myriad of metabolic and endocrine functional impairments. Hemodialysis patients continue to grapple with a constellation of ailments, including fatigue, hypotension, dietary constraints, lethargy, and an array of sexual and psychological challenges, such as anxiety and depression, which severely affect their overall well-being [7,8]. Consequently, their deteriorating health, concomitant shifts in psychological equilibrium, and limited capacity to fulfill social obligations collectively hamper their quality of life (QoL) [4].

These challenges, coupled with stringent dietary regimens, fluid restrictions, and heightened dependence on family members, can adversely affect the broader health-related QoL [7]. QoL is a pivotal parameter warranting meticulous assessment in the management of patients undergoing medical intervention [9]. It has been linked to elevated morbidity and mortality rates in this cohort, highlighting the need to consistently evaluate QoL as an integral component of healthcare outcomes [10,11]. Notably, Saudi Arabia’s Vision for 2020–2030 encompasses the enhancement of QoL for patients living with chronic illnesses [12], underscoring the crucial role that QoL plays in the comprehensive evaluation of long-term medical care outcomes [9]. As it is subjective in nature, QoL focuses on how a patient’s perceived health state affects their capacity to lead satisfying lives [13]. Numerous studies have revealed that hemodialysis patients with ESRD have a low QoL in Palestine [8], India [14], and Ethiopia [13]. However, determining QoL among hemodialysis patients offers a clear picture to provide more care and support for these patients.

Patients with ESRD have unique needs that offer invaluable insights into their illnesses, challenges, and treatment requirements. These needs empower them to take charge of their personal care and actively monitor their condition. Identifying and comprehensively assessing the needs of hemodialysis patients can lay the foundation for tailored educational programs attuned to their specific requirements, thereby enabling individuals to better manage their chronic illness while also enabling healthcare providers to devise individualized education initiatives tailored to the specific needs of each patient [15]. For instance, a study by Xhulia et al. (2016) in Greece revealed that hemodialysis patients expressed a significant need for information, guidance and support, trust in the medical and nursing staff, effective doctor/nurse communication, and assistance with both emotional and physical needs, underlining the multifaceted nature of their requirements. Similarly, a study conducted in Taiwan found that medical needs topped hemodialysis patients’ list of needs, closely followed by their physiological and psychological needs. These medical needs include understanding how to engage effectively with the medical staff and grasp the intricacies of their treatment plans [15,16].

This study offers a distinctive perspective by examining only the overall QoL as well as the specific determinants and nuanced relationships between QoL and the unmet needs of hemodialysis patients. Unlike prior research that primarily focuses on general QoL assessments, our study provides a granular analysis by identifying key psychosocial and clinical predictors that uniquely affect hemodialysis patients. To the best of our knowledge, the QoL and needs of hemodialysis patients, their intricate interrelationships, and the factors influencing them remain unexplored in the Ha’il region of Saudi Arabia. As such, this study aimed to ascertain the QoL and needs of hemodialysis patients while unraveling the relationships and predictive factors that may serve as a cornerstone for enhancing patient care. The findings hold the potential to inform the development of comprehensive strategies aimed at managing and ameliorating patient care.

## 2. Materials and Methods

### 2.1. Study Design, Setting, Population and Sample

A cross-sectional study design was employed, encompassing all patients with ESRD undergoing hemodialysis. This study was conducted at the primary dialysis center of King Khaled Hospital, which is situated in the Ha’il region of Saudi Arabia. This center provides dialysis services to patients with end-stage renal disease from various areas within the Ha’il region and has a capacity of 56 beds.

All patients who underwent hemodialysis between 15 January and 15 March 2023, and met the predefined study criteria were included.

**Figure 1:**
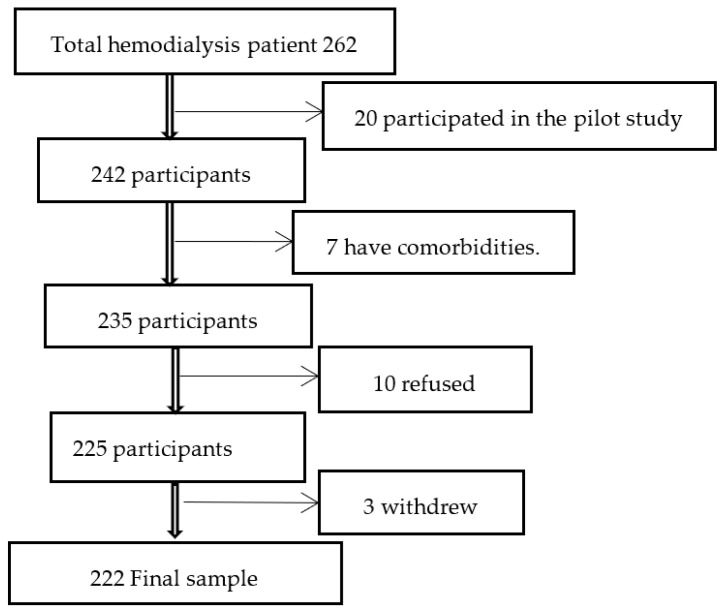
Flowchart depicting the sampling procedure.

Inclusion criteria: Patients aged 18 years or older, with a minimum of six months of regular hemodialysis, who consented to participate in the study. Exclusion criteria: Patients with cognitive impairment, intellectual disability, and comorbidities, such as congestive heart failure, stroke, chronic obstructive pulmonary disease, chronic liver disease, cancer, severe anemia, systemic lupus erythematosus, and uncontrolled diabetes mellitus, as these factors could act as confounding variables potentially affecting patients’ QoL and needs. The total sample size was 262 patients undergoing hemodialysis. Of these, 20 were excluded because they participated in the pilot study, 10 refused to participate, 3 withdrew their participation, and 7 were excluded due to comorbidities. Therefore, the final sample size was 222 (Figure 1).

### 2.2. Research Instruments

Structured interviews questionnaire was formulated based on prior research [16,17] and divided into three sections: sociodemographic characteristics, QoL, and patient needs.

Part I: Sociodemographic characteristics. This section gathered information on age, sex, educational level, marital status, current employment status, living arrangements, family support, income, body weight, height, causes of chronic kidney disease (CKD), duration and frequency of hemodialysis, and place of residence.

Part II: Quality of Life (QoL): QoL was assessed using the World Health Organization Quality of Life—Brief (WHOQOL-BREF) questionnaire, comprising 26 items. Two items pertained to the general aspects of QoL and health satisfaction, while twenty-four items spanned four domains—physical (seven items), psychological (six items), social (three items), and environmental (eight items)—to gauge patients’ QoL. Each question was rated on a five-point Likert scale with certain items in the physical (Q3 and Q4) and psychological (Q26) domains. QoL scores ranged from 24 to 120 points, with higher scores indicating better QoL [17].

Part III: Patient needs, developed by Xhulia et al., which consisted of 38 questions categorized into six domains: support/guidance needs (nine items), nurses and doctors’ informed needs (eight items), contact with other patient groups and maintaining communication with family members (six items), individualized treatment and personal participation needs (six items), physical-emotional needs (seven items), and nurses and doctors’ trust needs (two items). Responses were recorded on a four-point Likert scale with a range as follows: “very much = 4”, “very = 3”, “somewhat = 2”, and “not at all = 1”. Scores for patient needs range from 38 to 152 points, with higher scores indicating greater patient needs [16].

### 2.3. Validity and Reliability

The WHOQOL-BREF questionnaire, widely used to assess the QoL of CKD patients, was adopted in this study and has been validated in diverse settings [6,18,19].

The content validity index (CVI) for the patient needs tool was determined through evaluation by an expert panel comprising three nursing researchers with a Doctor of Philosophy (PhD), two nephrologists, and a PhD holder in Arabic, who assessed the questionnaire’s translation for clarity, readability, common terminology, and appropriateness. Panel members rated each item as “essential” (3), “useful but not essential” (2), or “unnecessary” (1). Both item CVIs (I-CVIs) and scale CVIs (S-CVIs) were calculated (values ranged from 0.83 to 1) to gauge the expert panel’s consensus on the relevance, clarity, and appropriateness of questionnaire items, with items that met the content validity criteria being retained.

A pilot study involving 20 patients was conducted to assess the item reliability and practicality. The findings indicated that the questionnaires were comprehensible and took approximately 15–20 min to complete. Internal reliability for the QoL and patient needs questionnaires, determined using Cronbach’s alpha, yielded values of 0.87 and 0.79, respectively, signifying high internal consistency.

### 2.4. Data Collection

Researchers have outlined the study objectives and data utilization for patients undergoing hemodialysis. Prior to the structured interviews, participants were informed of their right to withdraw or terminate their involvement without repercussions. To mitigate response bias, the researchers assured participants of response confidentiality and the importance of honest and accurate information. The complete anonymity of the responses was emphasized. Subsequently, hemodialysis patients voluntarily answered an anonymous questionnaire during structured interviews conducted during the postdialysis rest period. Anthropometric measurements, including height and dry weight, were obtained to calculate body mass index (BMI). The structured interviews lasted for approximately 10 min.

### 2.5. Data Analysis

All data were analyzed using Statistical Package for the Social Sciences software version 26 (SPSS, V26). Quantitative data were presented as means and standard deviations for normally distributed continuous variables, while categorical data were expressed as percentages and frequencies. A normality test was conducted for QoL and needs, confirming a *p*-value exceeding 0.05, indicating adherence to a normal distribution. Consequently, independent-samples *t*-tests were employed to explore associations between QoL and needs and various variables, including gender, living arrangements, residency, duration of hemodialysis, and frequency of hemodialysis per week. A one-way ANOVA was employed to determine the associations between QoL and needs and variables such as age, educational level, marital status, current employment status, family support, monthly income, BMI, and duration of hemodialysis (in hours). Pearson’s correlation was used to ascertain the strength of the direct relationship between QoL and needs domains. Multiple linear regression was used to identify the predictors of QoL and needs. The variables underwent multicollinearity testing using the variance inflation factor (VIF) and tolerance, with VIF values ranging from 1.2 to 2.8 and tolerance from 0.35 to 0.8, all falling below the accepted threshold of 5 [20], signifying an absence of multicollinearity concerns in the analysis. An alpha level of 0.05 was established as the statistical significance cutoff for the variables.

## 3. Results

### 3.1. Participants’ Characteristics, Hemodialysis History and Associatioen with QoL and Needs

#### 3.1.1. Participants’ General Characteristics

A demographic overview of the participants revealed that less than half (43.2%) of the participants fell within the age bracket of 41 to 60 years, with a mean age of 52.18 (SD = 16.60). More than half (51.8%) of the participants were male. Educational attainment varied, with 44.1% having graduated from secondary school or held a diploma and 28.4% having completed elementary school. The majority (64.9%) of participants were married. A significant proportion (64.4%) of participants were unemployed. In terms of residence, 71.6% of the respondents lived in residential areas. A substantial majority (92.8%) of them resided with their families. Approximately 41.4% reported full family support. The monthly income for the majority (70.7%) of the participants was less than 5000 Saudi Arabian riyals (SAR). Among the patients undergoing hemodialysis, 27.5% were classified as obese (Table 1).

#### 3.1.2. Hemodialysis History

Additional insights into the participants’ hemodialysis histories are presented in Table 1. The majority of the patients (51.4%) had undergone hemodialysis treatment for less than four years, with an average duration of 3.98 years. The vast majority (96.4%) of participants received hemodialysis three times per week. Regarding the duration of each hemodialysis session, 43.2% of the participants received treatment for four hours per session.

#### 3.1.3. Factors Associated with QoL and Needs of Patients with Hemodialysis

Table 1 provides a comprehensive overview of the factors associated with QoL and patient needs in patients undergoing hemodialysis. Significant differences were observed in the patients’ age, educational level, marital status, current employment status, living arrangements, family support, BMI, and hemodialysis duration (*p* < 0.05). Conversely, a higher mean of patient needs was observed among patients aged >60 years, females, those with no formal education, individuals who were divorced or widowed, retirees, those living alone, those lacking family support, those with an income of less than 5000 SAR, urban residents, individuals with a BMI ≥ 30, those who had been on hemodialysis for four years or longer, and those undergoing hemodialysis four times per week. Significant differences were noted in the patients’ age, sex, educational level, marital status, current employment status, monthly income, BMI, and hemodialysis duration (*p* < 0.05).

### 3.2. Overall QoL and Health Satisfaction

**Figure 2:**
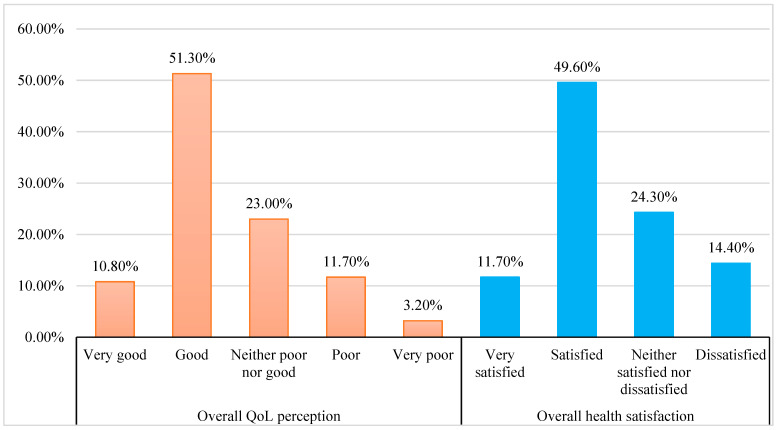
Overall QoL and health satisfaction.

The assessment of overall QoL and general health perception among hemodialysis patients yielded the following findings: A notable 51.30% of hemodialysis patients reported their QoL as “good”, while 10.80% described it as “very good”. In terms of health satisfaction, 49.60% of the patients reported being satisfied with their health (Figure 2).

### 3.3. Correlation Matrix for QoL and Needs Domains

High social and environmental QoL was negatively correlated with overall needs (*p* < 0.05). However, the need to trust the medical and nursing staff did not correlate significantly with the social domain (*p* = 0.132). Patients reporting a high psychological QoL displayed a reduced need for contact with other patient groups and had decreased requirements for emotional and physical support (*p* = 0.003 and 0.028, respectively). It is important to emphasize that none of the statistically significant correlations showed correlation coefficients reaching high values (−0.700 or 0.700). Overall, the trend indicated that patients with improved QoL tended to have fewer needs, as evidenced by the consistently negative correlation coefficients (*r* values) detailed in Table 2.

### 3.4. Factors Associated with QoL and Needs of Patients with Hemodialysis

Both multiple regression models for QoL and needs were statistically significant, as indicated by the adjusted R-squared values of 0.31 (F = 6.71, *p* < 0.001) for QoL and 0.17 (F = 3.82, *p* < 0.001) for needs.

#### 3.4.1. Predictors Associated with QoL

Hemodialysis patients aged 41–60 years demonstrated a QoL that was 14.71 points higher than that of patients over 60 years old (*t* = 4.75, *p* < 0.001, 95% CI = 8.60–20.81). Patients under 40 years of age had a QoL 7.77 points higher than that of patients over 60 years old. Married patients experienced a QoL 9.45 points higher than divorced or widowed patients (*p* = 0.008). points lower in patients living alone reported a QoL −10.39 points lower than in those living with their family (*t* = −2.19, *p* = 0.030, 95% CI = −19.75–−1.02). Patients with full family support had a QoL 8.83 points higher than those without (*t* = 3.05, *p* = 0.021, 95% CI = 1.33–16.33). Furthermore, patients with normal body weight or those classified as overweight had higher QoL (9.08 and 15.04 points, respectively) than obese patients. Patient needs significantly predicted QoL scores. Specifically, patients with higher needs have QoL lower by 0.11 points compared to those with lower needs (*t* = −2.37, *p* = 0.019, 95% CI = −0.20–−0.02). The most effective predictors of QoL were BMI between 25.0 and <30 kg/m^2^, being 41–60 years old, living alone (negative predictor), married, and BMI between 18.5% and <25 kg/m², with beta coefficients of 15.04, 14.71, −10.39, 9.45, and 9.08, respectively.

#### 3.4.2. Predictors Associated with Needs

Patients aged 41–60 years had needs scores that were 9.42 points lower than patients aged >60 years (*t* = −2.05, *p* = 0.041, 95% CI = −18.46–−0.37). Married patients exhibited lower needs scores (−16.95 points) than divorced or widowed patients (*t* = −3.39, *p* < 0.001, 95% CI = −26.80–−7.11). Patients with a normal BMI showed lower needs scores (−10.34 points) compared to those who were obese (BMI 30.0 or higher kg/m^2^; *t* = −2.44, *p* = 0.015, 95% CI = −18.69–−1.99). The most effective predictors of low patient needs were marital status (married), BMI between 18.5% and <25 kg/m^2^, and age between 41 and 60 years, with beta coefficients of −16.95, −10.34, and −9.42 respectively (Table 3).

## 4. Discussion

This study offers a comprehensive understanding of the needs and factors influencing QoL of hemodialysis patients in the Ha’il region of Saudi Arabia. The QoL of these patients can be significantly affected by deteriorating health, resulting in psychological changes and challenges in fulfilling social responsibilities. Importantly, QoL strongly influences health outcomes, and patients with lower QoL may face a poorer prognosis and declining health [6,11].

In some Arab countries, including Saudi Arabia, healthcare for hemodialysis patients has often focused primarily on physiological, clinical, and laboratory aspects [11,21] with insufficient attention given to patients’ psychological well-being [22]. Therefore, our study addresses a notable gap in knowledge regarding QoL and its determinants among hemodialysis patients in Saudi Arabia.

Interestingly, our study revealed that approximately two-thirds of hemodialysis patients reported good QoL and satisfaction with their health. This is in contrast to findings from other regions, such as Addis Ababa, Ethiopia, where only half of hemodialysis patients reported a positive QoL perception [11], and Greece, where the total QoL was 17.43 out of 30 [23]. Several factors might explain the relatively higher QoL among Saudi Arabian hemodialysis patients. Saudi Arabia’s cultural and religious beliefs, which emphasize acceptance of fate, even in the face of illness, may contribute to this positive outlook [4]. Additionally, the Ministry of Health in Saudi Arabia provides hemodialysis patients with special care, insurance, and transportation services, potentially alleviating their suffering and improving their quality of life. In a qualitative study conducted among Thai HD patients to identify their daily life experiences, three themes were identified: facing life limitations (e.g., physical inactivity), living with uncertainty (e.g., unknown future), and dependence on medical technology (e.g., inability to survive without HD machines) [24]. Despite the differences in the cultures of Saudi and Thai patients, these themes should be considered to improve their QoL [23].

A recent study highlighted a positive relationship between socioeconomic status and QoL in patients undergoing hemodialysis. This association can be attributed to the enhanced capacity of patients with better socioeconomic status to manage the financial burden associated with medications and other healthcare expenses during their illness [6]. However, the Saudi Arabian government bears all expenses related to the treatment of hemodialysis patients, which could potentially mitigate the impact of socioeconomic factors. Additionally, the quality of services offered at dialysis centers and the adequacy of the equipment provided may play pivotal roles in enhancing the QoL of patients undergoing hemodialysis. Notably, a study conducted in Rwanda demonstrated that hospitals with better healthcare services and improved access to care reported higher QoL among hemodialysis patients than other facilities. Conversely, research by Hasan et al. in Egypt indicated that inadequate hemodialysis services had a detrimental effect on patients’ QoL [22].

Nonetheless, our findings underscore the global importance of effective governance to ensure adequate and readily accessible healthcare for patients with chronic illnesses. An illustrative study conducted in Nepal revealed that hemodialysis patients reported lower perceived QoL across all domains of the WHOQOL-BREF, especially when compared to renal transplant recipients [6]. Nevertheless, existing research has consistently underscored the pivotal role of QoL as a critical indicator influencing clinical prognosis, recovery, and the overall quality of clinical care [25,26].

In summary, our study results underscore the significance of various sociodemographic factors in shaping the QoL of hemodialysis patients. Specifically, age, education level, work status, living arrangements, and family support were the main determinants. Notably, older hemodialysis patients in our study exhibited notably higher needs and lower QoL, particularly in the physical and social domains, compared to their younger counterparts. These findings align with prior research; Zyoud et al. reported similar results in Palestine [8], as did Alshraifeen et al. [27], Kim et al. [14], and Szu et al. in northern Taiwan [28]. Studies utilizing the same assessment tools, such as the one employed by Joshi et al., have found a positive association between the social domain and QoL in older adults [18], whereas Sathvik et al. did not observe an age-related association with all QoL domains [29]. Additionally, research conducted in Jordan indicated that older hemodialysis patients experienced more pronounced physical symptoms, psychological issues, and sleep disturbances than their younger counterparts [27]. Xhulia et al. (2016) further supported the notion that older adult patients have greater needs than their younger counterparts [16]. This could be attributed to the trend in Saudi Arabia, where older adults tend to engage in fewer physical activities [30], while younger adults shoulder multiple responsibilities based on their societal roles and positions. Furthermore, older patients may contend with a higher burden of physical, psychological, and social challenges related to immobility, whereas younger patients may experience shorter illness durations and fewer complications [8]. Consequently, our study underscores the pressing need for enhanced attention and care in older patients undergoing hemodialysis.

While our study revealed a higher average of associated needs among women than among men, it is important to note that there was no significant correlation between gender and QoL. This aligns with findings reported in studies conducted in Jordan [27] and Nepal [18]. Conversely, research conducted in Palestine [8] and Brazil [31] reported a lower QoL among female hemodialysis patients. It is worth mentioning that female hemodialysis patients in our study reported more negative somatic experiences, depression [27], and physical inactivity [32] than their male counterparts. One possible explanation for these sex-related differences could be that female Saudi patients predominantly reported patient needs associated with social isolation, limited community support, and challenges in engaging in physical activities, issues that are commonly observed in developing countries.

In terms of the sample’s educational background, our findings indicated that patients with higher levels of education reported significantly higher QoL and fewer needs, a trend consistent with a study conducted in Palestine [8]. This could be attributed to the fact that educated patients tend to have more information about their disease and its management than those with lower educational attainment. However, Joshi et al. (2017) found no discernible relationship between QoL and education level. This may suggest that highly educated patients have higher expectations for healthcare services, potentially resulting in reduced satisfaction and psychological well-being [18].

Marital status had a notable influence on both QoL and needs. In particular, married patients reported higher QoL than those who were unmarried, divorced, or widowed. This finding contradicts the results of a previous study conducted in Nepal [18]. However, this disparity might be attributable to the social support that married individuals typically receive from their families. This suggests the need for special attention from social workers and healthcare providers for patients in the latter category. Further research is warranted to gain a deeper understanding of the QoL experienced by these individuals.

Although our study revealed that unemployment and low monthly income appeared to be correlated with lower QoL scores, no statistically significant relationship was identified. However, unemployed individuals exhibited higher needs than their employed counterparts. These findings align with those reported in previous studies conducted in Palestine [8], Nepal [6,18], and Rwanda [33]. Shumbusho et al. also observed comparable associations, underscoring that employed patients tend to experience significantly better health-related QoL [33]. This observation can potentially be attributed to the influence of patients’ financial status and their capacity to meet societal demands, which play pivotal roles in enhancing QoL. Consequently, this study advocates the provision of support to these patients through the coverage of the expenses associated with their treatment.

Saudi society is known for robust family bonds and social relationships among its members. Our study confirmed this characteristic, as we found a significant relationship between higher QoL and patients who either lived with their families or received social support. These results underscore the significance of personal living conditions in shaping patient needs. Various factors, such as role changes, alterations in lifestyle, life limitations, economic and occupational prospects, and the ability to effectively communicate with significant individuals in their lives, contribute to these needs. These findings align with those of Nikkhah et al. [34].

Our study revealed that obese patients had significantly lower QoL and higher needs. These findings align with those of previous research; for example, Zyoud et al. reported a significant association between obesity and reduced QoL in Palestine [8]. The link between obesity and increased needs may be attributed to the elevated risk of comorbidities among obese patients, necessitating additional support to mitigate disease risk. Consequently, our results reinforce the recommendation of a prior study advocating the maintenance of an optimal weight in hemodialysis patients to enhance their QoL [35].

Our findings indicated no significant relationship between hemodialysis duration and QoL, which is consistent with previous studies [8,11,18]. Additionally, we observed a noteworthy association between patients’ place of residence and their QoL. Urban patients reported a higher QoL than rural patients, contrary to another study that reported no significant relationship between residency and QoL [8]. Shedding light on the myriad factors that influence QoL and the needs of hemodialysis patients can play an important role in enhancing their overall care. Many studies have underscored the significance of incorporating patient QoL and needs into the routine and ongoing assessment of healthcare outcomes, ensuring a holistic approach that addresses all facets of patient well-being [10,11].

The present study had several limitations. First, it was conducted at a single site and the sample size was relatively small. We included all eligible patients from the largest dialysis center in the Ha’il region, but did not incorporate data from private dialysis units or centers. Second, conducting face-to-face interviews during hemodialysis sessions may have influenced the patients’ responses. Third, we investigated QoL and patient needs independently of the effects of comorbidities. Future research is needed to elucidate the impact of comorbidities on QoL and patient needs. Fourth, this study employed a cross-sectional design and relied on self-reported measures. For future research, multicenter studies with large sample sizes and longitudinal designs are recommended along with interventions targeting the identified predictors. Finally, some questions, particularly those related to sexual activities, were potentially uncomfortable for the patients. Therefore, the generalizability of our results to the entire hemodialysis population may be limited.

## 5. Conclusions

This study revealed that over half of the hemodialysis patients reported having a good QoL, while less than half expressed satisfaction with their health. This highlights the need for interventions aimed at improving overall well-being and satisfaction among these patients. Importantly, patients with better QoL tended to have fewer needs, underscoring the complex interplay between various factors influencing QoL and needs. These factors included age, marital status, living status, and BMI. Furthermore, family support emerged as a significant contributor to QoL. Understanding these factors can aid in tailoring interventions to meet specific needs and improve the QoL in different patient groups. Sex and monthly income were also found to be associated with specific needs, emphasizing the importance of addressing sex-specific needs and challenges in hemodialysis care. These findings offer valuable insights for policymakers, healthcare providers, families, and the broader community to develop targeted strategies aimed at enhancing QoL and addressing the needs of individuals undergoing hemodialysis. This includes ensuring access to comprehensive healthcare services, addressing socioeconomic factors, promoting healthy lifestyles, and providing psychosocial support.

### Clinical Relevance

This study addressed a crucial aspect of healthcare by exploring the intricate relationship between QoL and the needs of patients undergoing hemodialysis. Hemodialysis is a lifeline for countless individuals with end-stage renal disease; however, their QoL remains a pressing concern. This study sheds light on this underexplored area and ultimately enhances the care provided to hemodialysis patients. This offers valuable insights that can inform both clinical practice and policy decisions, making it highly relevant to the field. These insights hold the potential to inform healthcare providers, policymakers, and researchers about strategies to improve the overall well-being of this patient population. Moreover, this research contributes valuable data to the broader international discourse on improving hemodialysis care, which can benefit patients worldwide.

## Figures and Tables

**Table 1:** Association between demographic characteristics, the hemodialysis history with QoL, and the needs of patients with hemodialysis (N = 222).

Demographic Characteristic	Overall F (%)	Quality of Life Mean ± SD	*p*-Value	Needs Mean ± SD	*p*-Value
Age **					
≤40 Years	60 (27.0)	77.01 ± 17.95	<0.0001	74.05 ± 22.29	0.014
41–60 Years	96 (43.2)	80.61 ± 15.58		79.21 ± 28.36	
>60Years	66 (29.7)	64.10 ± 23.34		87.81 ± 27.64	
Sex *					
Male	115 (51.8)	75.12 ± 20.40	0.766	74.80 ± 28.27	0.001
Female	107 (48.2)	74.31 ± 19.69		86.37 ± 24.38	
Educational Level **					
Illiterate	22 (9.9)	65.95 ± 21.74	0.009	98.09 ± 16.38	0.002
Primary school or below	63 (28.4)	72.80 ± 18.04		78.98 ± 26.55	
Secondary or diploma	98 (44.1)	74.64 ± 20.03		81.23 ± 28.03	
University and above	39 (17.6)	83.02 ± 19.81		70.48 ± 25.70	
Marital Status **					
Single	37 (16.7)	75.62 ± 16.56	0.002	76.40 ± 20.47	<0.001
Married	144 (64.9)	77.27 ± 19.13		76.04 ± 27.97	
Divorced/Widowed	41 (18.5)	65.02 ± 23.20		99.17 ± 20.51	
Current work status **					
Working	36 (16.2)	80.02 ± 23.20	0.018	68.47 ± 29.78	0.012
Not working	143 (64.4)	75.49 ± 18.66		83.34 ± 23.72	
Retired	43 (19.4)	67.76 ± 20.19		80.48 ± 32.47	
Living Status *					
Living Alone	16 (7.2)	65.06 ± 17.05	0.045	82.56 ± 26.56	0.738
Living with Family	206 (92.8)	75.48 ± 20.07		80.20 ± 27.13	
Family support **					
Full support	92 (41.4)	79.41 ± 22.51	0.010	77.71 ± 28.47	0.162
Partial support	91 (41.0)	72.27 ± 16.28		80.00 ± 25.15	
No support	39 (17.6)	69.43 ± 19.81		87.53 ± 27.24	
Monthly income **					
<5000 SAR	157 (70.7)	74.57 ± 17.87	0.953	84.00 ± 25.99	0.006
5000–10,000 SAR	53 (23.9)	74.83 ± 23.90		70.47 ± 28.21	
>10,000 SAR	12 (5.4)	76.41 ± 28.57		76.66 ± 25.83	
Residency *					
Urban	159 (71.6)	76.30 ± 19.73	0.063	80.94 ± 26.54	0.622
Rural	63 (28.4)	70.76 ± 20.35		78.95 ± 28.44	
Body Mass Index **					
Less than 18.5	8 (3.6)	73.75 ± 17.43	0.010	71.37 ± 23.47	0.041
18.5 to <25	99 (44.6)	76.64 ± 16.74		75.68 ± 25.18	
25.0 to <30	54 (24.3)	79.27 ± 13.02		82.50 ± 22.53	
30.0 or higher	61 (27.5)	67.73 ± 27.55		87.29 ± 32.33	
Hemodialysis period (years) *					
Less than 4	114 (51.4)	77.61 ± 19.16	0.027	76.78 ± 26.70	0.042
4	108 (48.6)	71.69 ± 20.54		84.16 ± 27.00	
Duration of hemodialysis (hours) **					
3	34 (15.3)	70.08 ± 19.73	0.072	73.05 ± 23.24	0.197
3.30	92 (41.4)	72.95 ± 22.68		82.82 ± 29.17	
4	96 (43.2)	78.08 ± 16.79		80.62 ± 25.95	
Frequency of hemodialysis (week) *					
3	214 (96.4)	75.30 ± 19.53	0.153	79.99 ± 27.03	0.270
4	8 (3.6)	59.50 ± 27.73		90.75 ± 26.78	

F, frequency. SD, standard deviation. *, *t*-test. **, ANOVA test.

**Table 2:** Correlation matrix for QoL and needs domains (N = 222).

Needs Domains		QoL Domains				
		Physical	Psychological	Social	Environment	Total QoL
Need for support and guidance	*r*	0.062	−0.011	−0.195 **	−0.136 *	−0.080
	*p*-value	0.360	0.870	0.004	0.043	0.236
Need to be informed by the medical and nursing staff	*r*	0.042	−0.096	−0.301 **	−0.258 **	−0.207 **
	*p*-value	0.538	0.155	<0.001	<0.001	0.002
Need for being in contact with other patient groups, and ensuring communication with relatives	*r*	−0.066	−0.199 **	−0.357 **	−0.329 **	−0.269 **
	*p*-value	0.328	0.003	<0.001	<0.001	<0.001
Need for individualized treatment and the patient’s personal participation in treatment	*r*	−0.057	−0.081	−0.272 **	−0.181 **	−0.154 *
	*p*-value	0.398	0.227	<0.001	0.007	0.022
Need for emotional and physical needs to be met	*r*	−0.026	−0.147 *	−0.220 **	−0.242 **	−0.187 **
	*p*-value	0.699	0.028	<0.001	<0.001	0.005
Need for trusting the medical and nursing staff	*r*	−0.009	−0.085	−0.101	−0.153 *	−0.112
	*p*-value	0.890	0.207	0.132	0.023	0.096
Total needs	*r*	0.013	−0.084	−0.264 **	−0.229 **	−0.160 *
	*p*-value	0.844	0.214	<0.001	<0.001	0.017

*r* = Pearson correlation coefficient, ** significant at the 0.01 level; *, significant at the 0.05 level.

**Table 3:** Multiple linear regression analysis for predicting factors affecting QoL and needs of patients with hemodialysis (N = 222).

Factors	Quality of Life				Needs			
	B *	*t*	Sig.	95% CI	B *	*t*	Sig.	95% CI
Age								
≤40 years	7.77	1.88	0.062	−0.40–15.93	−5.70	−0.93	0.351	−17.73–6.32
41–60 years	14.71	4.75	<0.001	8.60–20.81	−9.42	−2.05	0.041	−18.46–−0.37
>60 years	reference				reference			
Sex								
Female					7.31	1.695	0.092	−1.19–15.81
Male					reference			
Educational Level								
Illiterate	−1.73	−0.30	0.766	−13.18–9.72	6.33	0.74	0.459	−10.51–23.17
Primary school or below	−7.03	−1.68	0.095	−15.29–1.23	2.79	0.45	0.648	−9.27–14.87
Secondary or Diploma	−3.05	−0.82	0.415	−10.40–4.31	5.23	0.93	0.349	−5.77–16.24
University and above	reference				reference			
Marital Status								
Single	3.46	0.68	0.497	−6.56–13.49	−12.78	−1.71	0.088	−27.48–1.91
Married	9.45	2.68	0.008	2.49–16.40	−16.95	−3.39	<0.001	−26.80–−7.11
Divorced/Widowed	reference							
Current work status								
Not working	0.84	0.22	0.829	−6.82–8.50	8.49	1.35	0.178	−3.89–20.87
Retired	−8.34	−1.84	0.068	−17.28–0.61	10.03	1.49	0.136	−3.18–23.25
Working	reference				reference			
Living Status								
Living alone	−10.39	−2.19	0.030	−19.75–−1.02				
Living with family	reference							
Family support								
Full support	8.83	2.32	0.021	1.33–16.33				
Partial support	−0.17	−0.05	0.963	−7.38–7.03				
No support	reference							
Monthly income								
<5000 SAR					−9.33	−1.07	0.282	−26.39–7.72
5000–10,000 SAR					−16.71	−1.90	0.058	−34.02–0.58
>10,000 SAR					reference			
Body Mass Index								
Less than 18.5 kg/m^2^	7.62	1.13	0.258	−5.62–20.86	−15.00	−1.54	0.123	−34.10–4.08
18.5 to <25 kg/m^2^	9.08	3.05	0.003	3.22–14.93	−10.34	−2.44	0.015	−18.69–−1.99
25.0 to <30 kg/m^2^	15.04	4.43	<0.001	8.34–21.74	−2.79	−0.57	0.564	−12.31–6.73
30.0 or higher kg/m^2^	reference				reference			
Hemodialysis period								
Less than 4 years	1.76	0.69	0.492	−3.29–6.82	−3.49	−0.92	.355	−10.92–3.93
4 years or higher	reference				reference			
Patient Needs	−0.11	−2.37	0.019	−0.20–−0.02				

B *, Unstandardized Coefficients B; QoL model: R square = 0.36, adjusted R square = 0.31, F = 6.71, *p* < 0.001; patient needs model; R square = 0.23, adjusted R square = 0.17, F = 3.82, *p* < 0.001.

## Data Availability

Data are available upon request from the corresponding author.
